# The influence of functional groups and their relative position on isotopic exchange between ^19^F and ^18^F in aromatic rings

**DOI:** 10.1186/s41181-026-00494-4

**Published:** 2026-08-31

**Authors:** Antonia A. Högnäsbacka, Miguel A. Cortés González, Afrizki D. Wiranegara, Magnus Schou

**Affiliations:** 1 Department of Clinical Neuroscience, Centre for Psychiatry Research, Karolinska Institutet and Stockholm County Council, Stockholm, Sweden; 2 Radiology and Theranostics, Cancer Biomarker Development, Early Oncology R&D, AstraZeneca, Stockholm, Sweden

**Keywords:** Isotopic exchange, Halogen exchange, Nucleophilic aromatic substitution, Fluorine-18, PET, Fluorobenzene

## Abstract

**Background:**

Isotopic exchange with fluorine-18 offers a conceptually simple approach to positron emission tomography (PET) tracer development, as the target compound itself serves as the labeling precursor.

**Results:**

In this study, we investigated the influence of functional groups and substituent position on the efficiency of isotopic exchange with fluorine-18. A total of 19 fluorobenzene derivatives were screened under standardized conditions, in dimethyl sulfoxide (175 °C) and dimethylformamide (150 °C) as solvents. Only a subset of compounds underwent efficient exchange; electron-withdrawing substituents strongly promoted isotopic exchange, with ortho and para isomers generally being more reactive than meta-analogues. The kinetics of the exchange in dimethyl sulfoxide were quantified for fluorobenzonitriles, nitrofluorobenzenes, and fluorobenzaldehydes. Nonlinear regression analysis indicated more rapid apparent exchange for the ortho- and para-substituted nitrile and nitro derivatives, together with higher apparent plateaus (≥84–94%), consistent with an S_N_Ar-like mechanism involving stabilization of the Meisenheimer intermediate. By contrast, meta isomers were significantly less reactive, and no exchange was detected for 3-fluorobenzaldehyde. Fluorobenzaldehydes also showed lower overall apparent plateaus and signs of thermal instability in dimethyl sulfoxide. Full-scale labeling confirmed the feasibility of high molar activity, with isolated 2-[^18^F]fluorobenzonitrile obtained at 121 ± 37 GBq/µmol. Although radiochemical yield (5.84±2.23%, decay-corrected to the addition of tetraethylammonium [^18^F]fluoride) and radiochemical purity (88.6 ± 3.9%) remained modest, these results demonstrate that isotopic exchange can provide molar activities within the range required for PET radiopharmaceuticals.

**Conclusions:**

These findings clarify the functional group and positional influences on fluorine-19/fluorine-18 isotopic exchange, highlighting the potential of this strategy to streamline PET tracer development by circumventing the need for precursor development and extensive reaction optimization.

**Supplementary Information:**

The online version contains supplementary material available at https://doi.org/10.1186/s41181-026-00494-4.

## Background

Positron emission tomography (PET) is an imaging technique that utilizes the decay of radioactive isotopes to visualize real-time three-dimensional biodistribution of compounds (Ametamey, Honer and Schubiger 2008; Jones and Townsend 2017; Phelps 2000). Fluorine-18 is widely used in PET imaging due to its ability to substitute fluorine-19 in a molecule, enabling the synthesis of radiolabeled analogues that are chemically and biologically indistinguishable from their non-radioactive counterparts. The presence of fluorine in drug molecules is common, as the introduction of one or more fluorine atoms can enhance both metabolic stability and bioavailability (Böhm et al. 2004; Müller, Faeh and Diederich 2007; Purser et al. 2008). Fluorine-18 is highly suitable for radiolabelling of small molecule PET tracers, as it has a moderate half-life of about 109.7 min, and a high β^+^ decay fraction (97%) (National Nuclear Data Center NNDC 2025).

There are two fundamental ways of covalently introducing fluorine-18 in a molecule: electrophilic fluorination and nucleophilic fluorination. Electrophilic fluorination is a chemical reaction in which an electrophilic fluorine source is used to introduce a fluorine atom into an electron-rich site of an organic molecule. The technique is limited by low specific activity stemming from the [^18^F]F_2_ production, its high reactivity, poor functional group tolerance, poor regioselectivity, high oxidizing power, corrosiveness, and the limited availability of [^18^F]F_2_. Nucleophilic fluorination is a chemical reaction in which a nucleophilic fluorine source (typically the fluoride ion, [^18^F]F^−^) displaces a leaving group in an organic molecule, introducing a fluorine atom in a site-specific manner. The method is limited by the requirement for a precursor designed for the purpose, and often harsh reaction conditions, which can hinder late-stage fluorination of complex molecules. Isotopic exchange is a method where the [^18^F]fluoride exchanges with a non-radioactive ^19^F atom already present in the substrate, forming a radiolabeled product that is chemically identical to the original compound. Isotopic exchange, predominantly carried out using nucleophilic conditions, is limited by low molar activity and applicability to substrates with sufficiently electron-deficient fluorinated positions that can undergo nucleophilic exchange, with the required reaction conditions depending strongly on substrate reactivity (Cole et al. 2014; Wang et al. 2023).

While high molar activity is often critical for specific imaging of low-density or low-abundance targets, certain applications allow for a lower molar activity either because the target isn’t saturable in the relevant range, or the endogenous substrate is in huge excess. Under these circumstances, isotopic exchange provides an attractive alternative for compounds containing fluorine. In the context of aromatic isotopic exchange, studies have demonstrated that the incorporation of strategically chosen functional groups can significantly enhance the fluorine-19/fluorine-18 exchange by modulating the electronic properties of the ring structure (Blom, Karimi and Långström 2009; Shen et al. 2007). The activating groups can also be designed as temporary handles that can be converted into alternative functionalities, enabling access to chemically diverse targets without compromising the radiochemical yield. The present work investigated how functional groups promote or hinder isotopic exchange via nucleophilic aromatic substitution, and how substitution patterns influence its efficiency. In addition to evaluating functional group and positional effects on isotopic exchange, we also assessed the ability of the method to achieve molar activities comparable to those typically obtained in conventional fluorine-18 radiolabeling.

## Methods

Under standardized screening conditions, isotopic exchange with [^18^F]fluoride was performed on 19 compounds (Table S1) to assess the influence of different functional groups on promoting or hindering the exchange, as well as the positional effects of substituents on the isotopic exchange. The screening set consisted of fluorobenzene derivatives bearing a variety of functional groups, including mono-, di-, and trifluorobenzene derivatives, as well as fluorobenzene derivatives bearing nitro, nitrile, aldehyde, methoxy, and methyl substituents in different positions.

Several parameters must be considered when designing fluorine-18 reactions, beginning with the trapping, releasing and drying of the fluorine-18. Radiolabeling with fluorine-18 usually begins with the isolation of the [^18^F]fluoride from the [^18^O]water using a solid-phase extraction cartridge pre-conditioned with a carbonate or bicarbonate solution. The [^18^F]fluoride is subsequently released from the solid-phase extraction cartridge using a suitable eluent, commonly potassium carbonate in combination with a cryptand, such as Kryptofix 2.2.2, or alternatively tetrabutylammonium bicarbonate. The method used for [^18^F]fluoride preparation can have a significant impact on the subsequent reaction.

In this study, [^18^F]fluoride was isolated using an Oasis WAX (weak anion exchange) solid-phase extraction cartridge, conditioned with sodium bicarbonate. Elution was achieved using tetraethylammonium bicarbonate for two reasons: (i) bicarbonate provides a milder basic environment than carbonate, reducing the risk of substrate degradation, (ii) avoiding the use of cryptands simplifies the purification of the final product and eliminates the risk of residual chelators.

### [^18^F]fluoride production, trapping and elution

[^18^F]Fluoride was produced in a PETtrace 880 (GE Healthcare, Uppsala, Sweden) cyclotron by irradiating 2.7 mL of [^18^O]water (Taiyo Nippon Sanso, Tokyo, Japan). For the screening experiments, rinse water from clinical productions (e.g. [^18^F]FDG) was often used. When required, the target was irradiated at 15 μA for 2–6 min, depending on the residual activity and number of experiments performed that day. For molar activity experiments, the target was irradiated at 35 µA for 3.5 min.

The [^18^F]fluoride processing was completed in a custom-built synthesis module (DM Automation, Nykvarn, Sweden). The [^18^F]fluoride was separated from the [^18^O]water using an anion exchange Oasis® WAX 1cc 30 mg cartridge (Waters Corporation, Milford, Massachusetts USA) conditioned with sodium bicarbonate (aq. 0.5 M, 3 mL) and subsequently Milli-Q water (3 mL). The [^18^F]fluoride was released from the solid phase extraction cartridge by elution with a freshly prepared tetraethylammonium bicarbonate solution (1 mL, 0.01 M in 97:3 acetonitrile/Milli-Q water). The eluted tetraethylammonium [^18^F]fluoride was azeotropically dried by heating the vial to 110 °C under a flow of nitrogen (40 mL/min) during 10 min before cooling the vial and adding 400 μL of acetonitrile and repeating the drying procedure.

### Substrate preparation

Substrate stock solutions were prepared by transferring the substrate, either by pipetting or weighing, into a 4 mL flat-bottomed vial and adding anhydrous solvent to obtain a 0.5 µmol/µL stock solution. For the concentration-dependency experiments, lower substrate concentrations were prepared by stepwise dilution of a 0.5 µmol/µL stock solution in anhydrous dimethyl sulfoxide. All solutions were stored at −20 °C and thawed and vortexed prior to use.

### Screening conditions

Anhydrous solvent (4 mL) was added to the vial containing dried tetraethylammonium [^18^F]fluoride, followed by vortexing. An aliquot of tetraethylammonium [^18^F]fluoride (190 µL, approximately 29–57 MBq) was then transferred into a 4 mL flat-bottomed vial containing substrate stock solution (10 µL, 5 µmol). For the concentration-dependency experiments, the same procedure was used with substrate amounts ranging from 0.5 nmol to 5 µmol in 10 µL and 18–19 MBq of tetraethylammonium [^18^F]fluoride per reaction. The reaction mixture was heated at 175 °C (for dimethyl sulfoxide) or 150 °C (for dimethylformamide) for 20 minutes, after which it was cooled at room temperature for 1 minute before the reaction was quenched by the addition of water (0.1 mL).

### Kinetic experiments

Anhydrous dimethyl sulfoxide (4 mL) was added to the vial containing dried tetraethylammonium [^18^F]fluoride, followed by vortexing. An aliquot of tetraethylammonium [^18^F]fluoride (1140 µL) was then transferred into a 4 mL flat-bottomed vial containing substrate stock solution (60 µL). The reaction mixture was then vortexed and divided into six 4 mL flat-bottomed vials (200 µL/vial). The reaction mixture was heated at 175 °C for the specified time, after which it was cooled at room temperature for 1 minute before the reaction was quenched by the addition of water (0.1 mL). The kinetic calculations were carried out using GraphPad Prism (version 10.6.0). Conversion-time data were fitted by nonlinear least-squares regression to a one-phase association model.

### Scaled-up concentration-dependency experiments

Anhydrous dimethyl sulfoxide (190 µL) was added to the vial containing dried tetraethylammonium [^18^F]fluoride (629–965 MBq), followed by vortexing. The solution was then transferred into a 4 mL flat-bottomed vial containing 2-fluorobenzonitrile solution (10 µL, 5 nmol–5 µmol), giving a total reaction volume of 200 µL. The reactions were otherwise performed as described under the screening conditions.

### Analysis

The radiochemical conversion was determined by analytical HPLC (radio-detection) of the crude reaction mixture using an Agilent 1220 Infinity LC (California, US) equipped with a Flow-Count 2Ch radiodetector (Eckert & Ziegler Radiopharma Inc, Massachusetts, USA), using an XBridge BEH C18 column (150 × 4.6 mm, 5 μm, Waters Corporation, Massachusetts, USA) and acetonitrile in 0.1 M ammonium formate (aq.). Either the HPLC method gradient M1 (20% for 1 min; 20 to 50% in 5 min; 50% held for 2 min; 50 to 20% in 1 min; at 2 mL/min; UV detection wavelength of 254 nm) or M2 (40% for 1 min; 40 to 80% in 5 min; 80% held for 2 min; 80 to 40% in 1 min; at 2 mL/min; and UV detection wavelength of 254 nm) was used, with the choice of method determined by compound-specific retention behaviour as outlined in Table 1.

**Table 1:** HPLC method definition for each compound to determine the radiochemical conversion

Compound	HPLC method	Compound	HPLC method
1,2-difluorobenzene	M1	3-fluorobenzaldehyde	M1
1,3-difluorobenzene	M1	4-fluorobenzaldehyde	M1
1,4-difluorobenzene	M1	fluorobenzene	M2
2-fluorobenzonitrile,	M1	1,2,3-trifluorobenzene	M2
3-fluorobenzonitrile	M1	3-fluoroanisole	M2
4-fluorobenzonitrile	M1	4-fluoroanisole	M2
1-fluoro-2-nitrobenzene	M1	2-fluorotoluene	M2
1-fluoro-3-nitrobenzene	M1	3-fluorotoluene	M2
1-fluoro-4-nitrobenzene	M1	4-fluorotoluene	M2
2-fluorobenzaldehyde	M1		

### Molar activity experiments

Anhydrous dimethyl sulfoxide (190 µL) was added to the vial containing dried tetraethylammonium [^18^F]fluoride, followed by vortexing. The solution was then transferred into a 4 mL flat-bottomed vial containing 2-fluorobenzonitrile solution (10 µL of 0.5 nmol/µL). The reaction mixture was heated at 175 °C for 20 minutes, after which it was cooled at room temperature for 3 minutes before the addition of MilliQ water (2 mL). Using the synthesis module, 2-[^18^F]fluorobenzonitrile was isolated using an OASIS®HLB 3cc 60 mg solid-phase extraction cartridge (Waters Corporation, Milford, Massachusetts USA, conditioned with 3 mL ethanol followed by 3 mL water). The vial and solid-phase extraction cartridge were rinsed with MilliQ water (2 × 4 mL). The solid-phase extraction cartridge was then detached from the module and hand-eluted with ethanol (1 mL). The product was weighed, and the radioactivity was measured on a CRC-120 radioisotope calibrator (Capintec, now Mirion Technologies, New Jersey, USA). The molar activity was determined using an XBridge BEH C18 column (150 × 4.6 mm, 5 μm, Waters Corporation, Massachusetts, USA) and 40% acetonitrile in 0.1 M ammonium formate (aq.), at 2 mL/min. UV detection was performed at 224 nm, and the peak area was quantified using a three-point calibration curve. The calibration curve was linear in the range 0.5–50 nmol/mL (R^2^ > 0.99). The molar activity was calculated by correcting the measured radioactivity of the product for radiochemical purity as determined by HPLC. Radiochemical yield was decay-corrected to the addition of tetraethylammonium [^18^F]fluoride solution to the reaction. An exemplary HPLC chromatogram is presented in the supplementary information (Figure S1).

## Results

Isotopic exchange with [^18^F]fluoride was investigated for a set of 19 substituted fluorobenzenes under standardized screening conditions to evaluate the influence of functional groups and substitution patterns on the efficiency of exchange. The isotopic exchange in dimethyl sulfoxide at 175 °C revealed that compounds bearing nitrile, nitro, and aldehyde substituents underwent efficient labeling, confirming their compatibility with the exchange conditions (Table 2). Trifluorobenzene showed moderate reactivity, with radiochemical conversion reaching up to 45%, while difluorinated analogs exhibited only minimal exchange. In contrast, electron-donating groups such as methyl and methoxy resulted in no detectable conversion. Positional effects were also pronounced: ortho-substituted substrates consistently showed the highest labeling efficiency, followed by para-substituted substrates, whereas meta-substituted compounds performed worse.

**Table 2:**
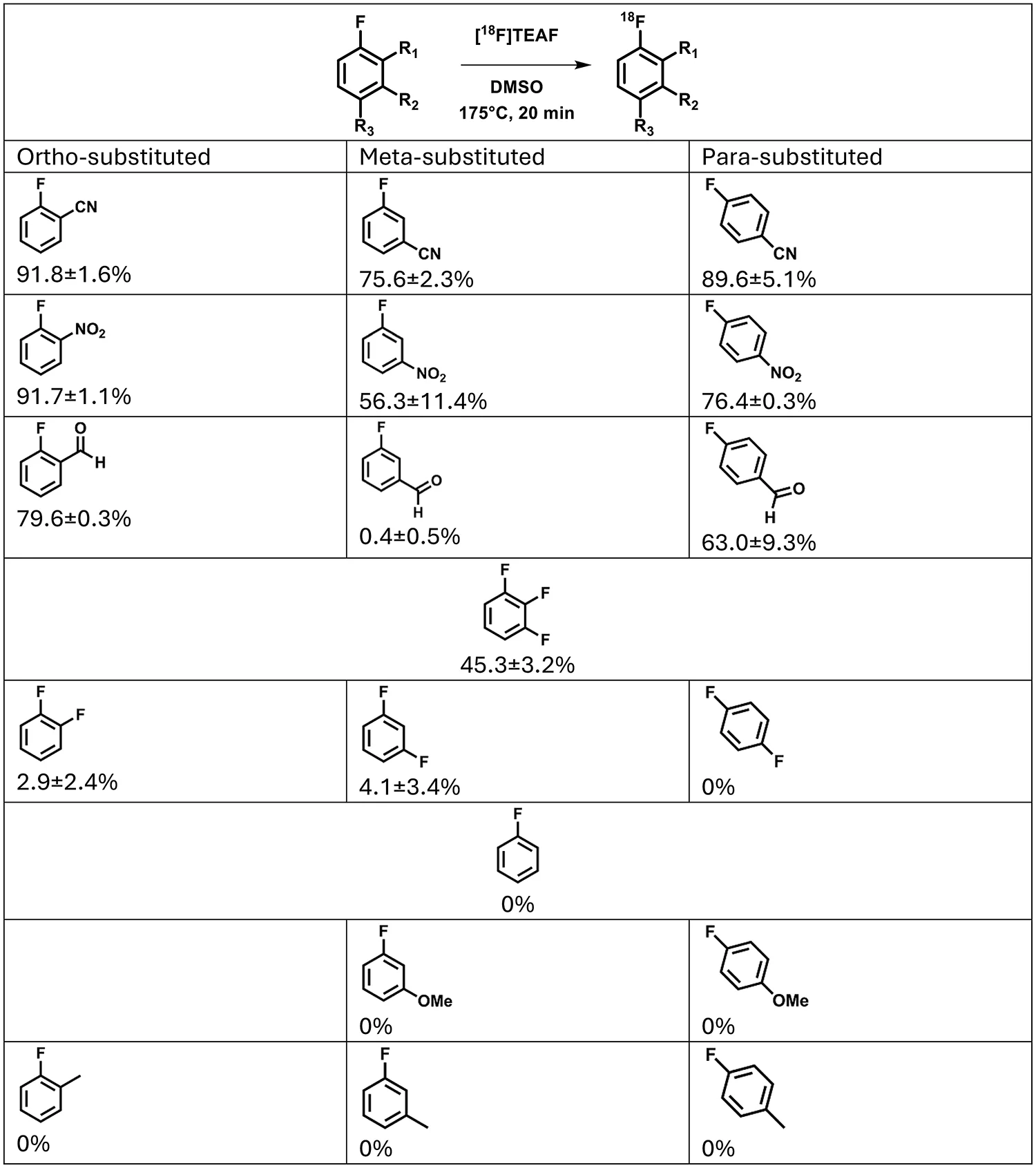
Isotopic exchange screening of substituted fluorobenzenes (25 mM, *n* = 3) under standardized conditions in DMSO

For comparison, the screening was repeated in dimethylformamide at 150 °C, adjusted to account for the lower boiling point (Table 3). Under these conditions, the overall conversion was significantly reduced across all compounds, highlighting the importance of the reaction conditions for facilitating efficient isotopic exchange. The previously observed trend in positional reactivity (ortho > para >meta) was not preserved in the second screening.

**Table 3:**
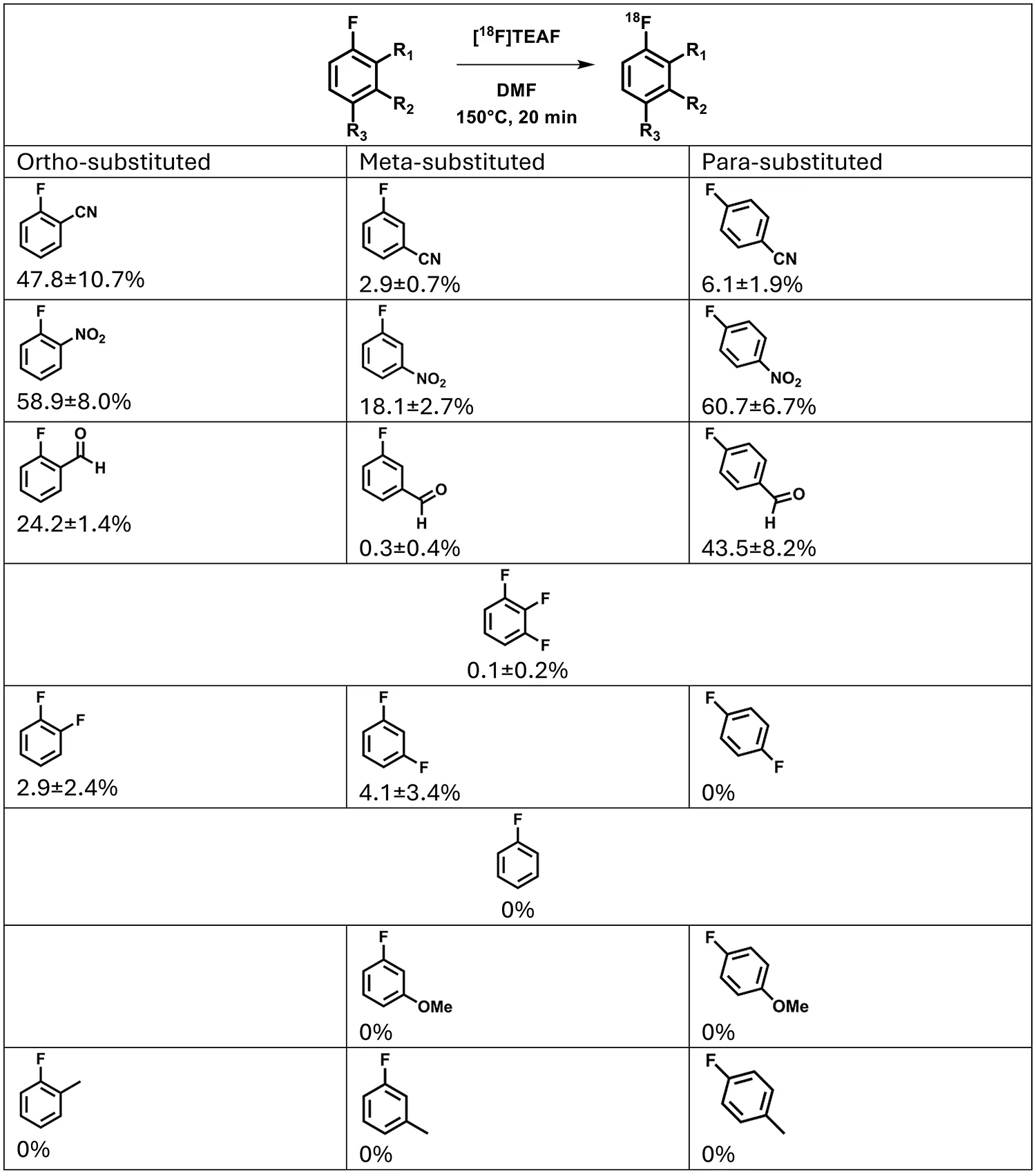
Isotopic exchange screening of substituted fluorobenzenes (25 mM, *n* = 3) under standardized conditions in DMF

**Fig. 1:**
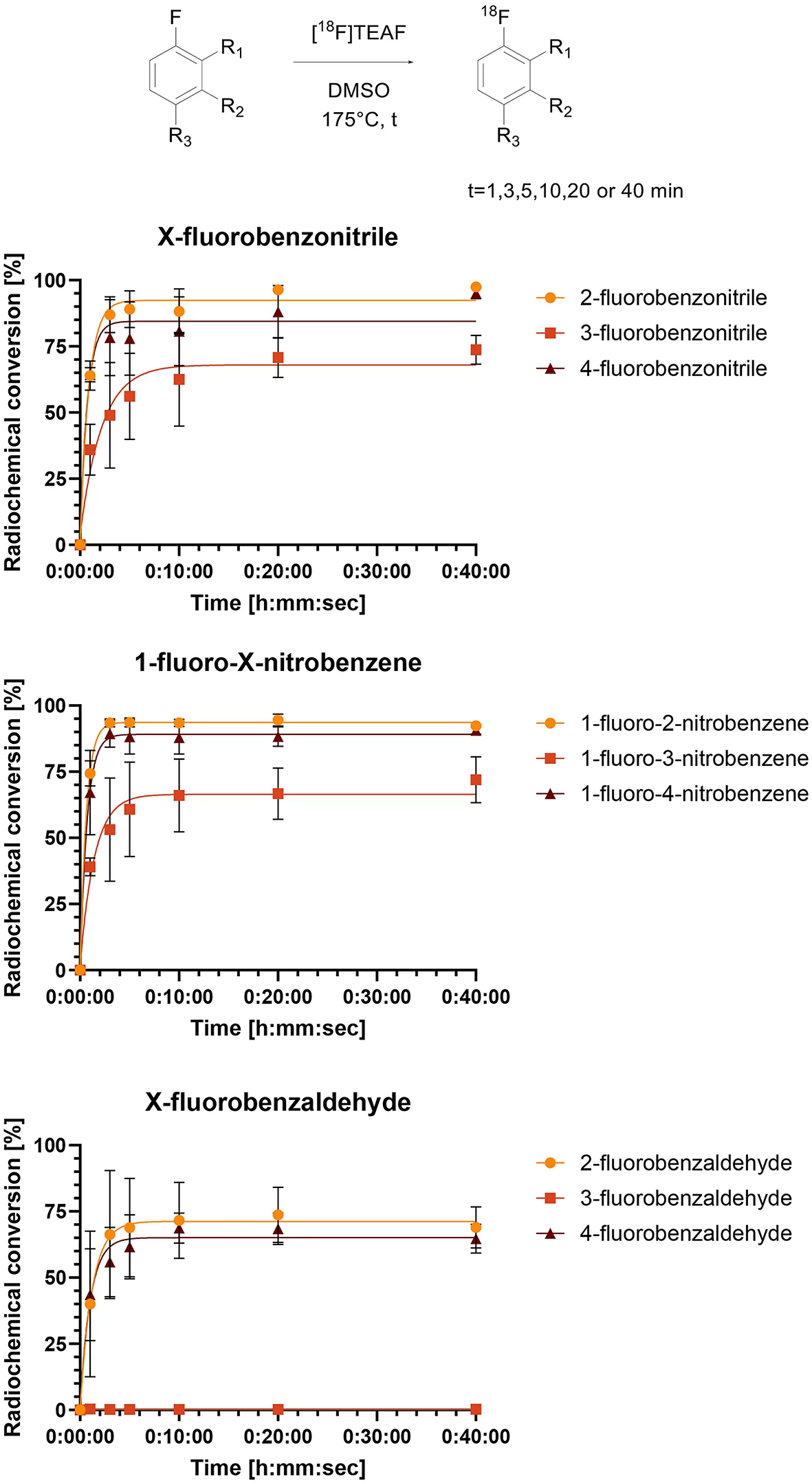
Kinetics of isotopic exchange of substituted fluorobenzenes in DMSO (175 °C). Conversion–time data (1–40 min) are shown for fluorobenzonitriles, nitrofluorobenzenes, and fluorobenzaldehydes, with nonlinear regression fits (one-phase association) overlaid. Error bars represent mean ± SD (*n* = 3)

Based on the initial screening, only a selection of the 19 compounds showed efficient isotopic exchange. To gain deeper insight into the factors governing this reactivity, we next examined the apparent kinetics of isotopic exchange for fluorobenzonitriles, nitrofluorobenzenes and fluorobenzaldehydes (Fig. 1). Due to the experimental variability associated with fluorine-18 (e.g. drying efficiency and fluoride availability), and the sampling intervals used, the kinetic parameters reported here should be regarded as apparent parameters that describe relative trends across substrates rather than intrinsic rate constants. Nonlinear regression of conversion–time data in dimethyl sulfoxide (at 175 °C) indicated clear substituent and positional effects (Table S2). Ortho- and para-nitrofluorobenzenes showed the most rapid exchange (*o*-NO₂: k_obs_ = 1.59 min^−1^, t_1/2_ = 0.44 min; *p*-NO₂: 1.41 min^− 1^, t_1/2_ = 0.49 min), with high apparent plateaus (89–94%). Fluorobenzonitriles showed a similar pattern, with more rapid exchange at ortho and para positions (k_obs_ = 1.14–1.38 min^−1^, t_1/2_ = 0.50–0.61 min), but as for nitrofluorobenzene, meta exchange was slower (0.46 min^− 1^, t_1/2_ = 1.52 min) and plateaued at only 68%. In contrast, the fluorobenzaldehydes exhibited lower apparent plateaus overall (65–71%), and greater variability in the kinetic profiles, reflected in wide confidence intervals for k_obs_. The meta isomer did not undergo measurable exchange under the screening conditions. In addition, qualitative observations suggested degradation of the fluorobenzaldehydes when heated in dimethyl sulfoxide, which was not apparent in dimethylformamide, consistent with the findings of Shen et al. (2007), potentially complicating the interpretation of these results.

**Fig. 2:**
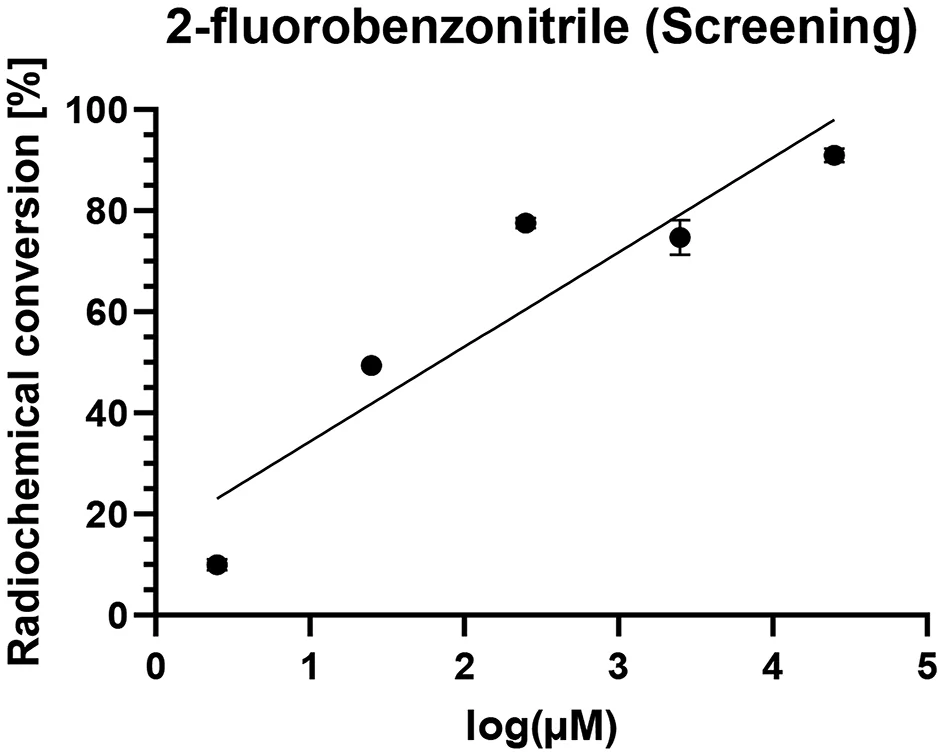
Concentration dependence of radiochemical conversion for 2-fluorobenzonitrile under screening conditions. Data were fitted by linear regression (R^2^ = 0.85, *n* = 2) to log-transformed concentration values. Raw data underlying the fit are provided in the supplementary information

Based on these considerations, 2-fluorobenzonitrile was chosen for subsequent molar activity experiments, as it combined efficient and reproducible exchange behavior with the synthetic versatility of the nitrile group (Rakshit et al. 2022; Xia, Jiang and Wu 2021), making it a representative substrate for further evaluation. Since the isotopic exchange of nitrile-containing compounds proceeded efficiently, we further investigated the labelling of 2-fluorobenzonitrile by systematically lowering the substrate amount in order to assess the achievable molar activity. The substrate load was reduced from the 25 mM used in the screening experiments down to 2.5 µM (Fig. 2, Table S3).

**Fig. 3:**
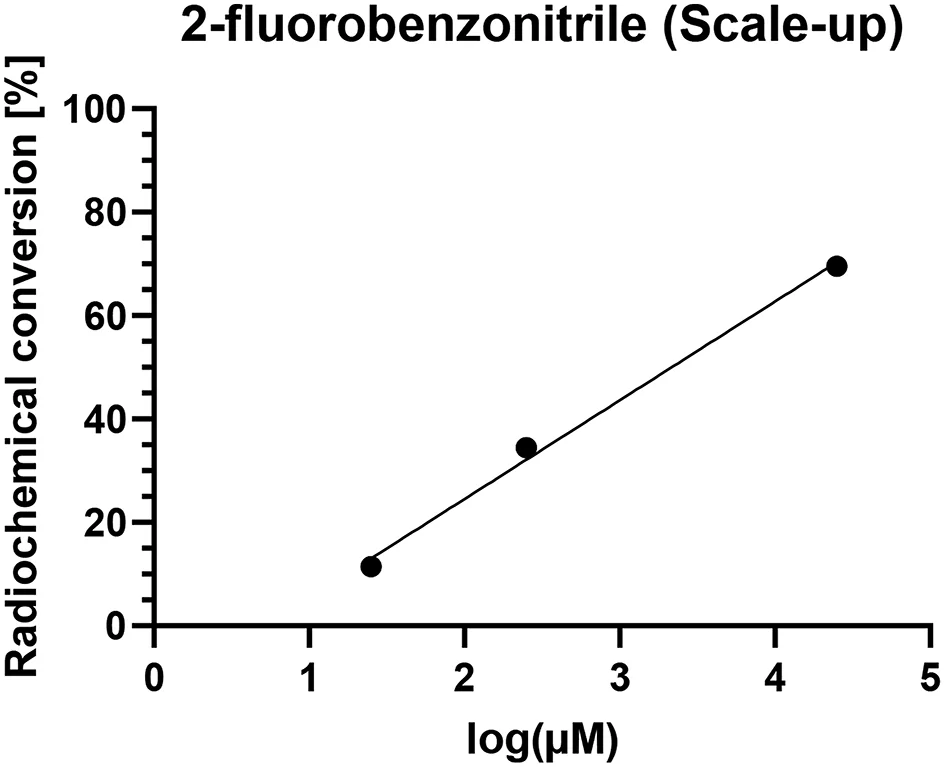
Concentration dependency on radiochemical conversion for 2-fluorobenzonitrile under scale-up conditions. Data were fitted by linear regression (R^2^ = 0.99, *n* = 1). Raw data underlying the fit are provided in the supplementary information

At the lowest substrate concentration tested (2.5 µM) a radiochemical conversion of about 10% was achieved. If the conversion was maintained under scale-up conditions, and 2 GBq had been used instead of the 20 MBq applied under the screening conditions, a molar activity of ~ 400 GBq/µmol could have theoretically been obtained. This finding prompted further evaluation under scaled-up reaction conditions. The reaction was scaled up by increasing the radioactivity to approximately 1 GBq, together with a 20-fold increase in tetraethylammonium bicarbonate (Fig. 3, Table S4).

At 25 mM, the radiochemical conversion decreased from approximately 90% under screening conditions to approximately 70% under scale-up conditions. At 25 µM a more pronounced reduction was observed with the conversion decreasing from approximately 50% under screening conditions to approximately 10% under scale-up conditions. Overall, these results indicate a substantially reduced radiochemical conversion under scale-up conditions. Since this experiment was intended only to assess the feasibility of achieving high molar activity, no efforts were made to optimize the radiochemical conversion or to further assess reaction parameters. Instead, the lowest substrate concentration in the scaled-up concentration dependency experiment (25 µM) was combined with higher radioactivity, and the product was isolated using a solid-phase extraction cartridge to enable the determination of the molar activity of the isolated product. Using 3.50 ± 0.25 GBq of tetraethylammonium [^18^F]fluoride, the isotopic exchange of 2-fluorobenzonitrile yielded 166.67 ± 78.36 MBq of isolated 2-[^18^F]fluorobenzonitrile, corresponding to a molar activity of 121.39 ± 36.50 GBq/µmol (Table S5). The radiochemical yield was 5.84 ± 2.23% (decay-corrected to the addition of tetraethylammonium [^18^F]fluoride), and the radiochemical purity was 88.55 ± 3.90%. Radio-HPLC analysis consistently showed two early-eluting radioactive impurities in addition to the product peak (Supporting Information, Figure S1). Their identity was not investigated in the present study. The isolated 2-fluorobenzonitrile concentration did not match the theoretical amount of 2-fluorobenzonitrile added to the reaction mixture. This may be related to volatilization during the high-temperature reaction, as slight deformation of the reaction caps was observed after heating to 175 °C, although this possibility was not investigated experimentally.

## Discussion

This study aimed at investigating how functional groups and their position influence the efficiency of fluorine-19/fluorine-18 isotopic exchange. Nineteen substituted fluorobenzenes were systematically screened, and the kinetics of the isotopic exchange were further assessed for a subset of compounds. Lastly, the molar activity for one of the compounds was evaluated.

Isotopic exchange occurred most efficiently in compounds bearing strong electron-withdrawing groups such as nitro and nitrile substituents when reacting in dimethyl sulfoxide. Within these classes, ortho and para isomers showed fast exchange rates and high conversion plateaus, while meta isomers were consistently slower and reached lower plateaus. These results are consistent with an S_N_Ar-type mechanism (Fernández, Frenking and Uggerud 2010; Terrier 1982), where electron-withdrawing groups increase the electrophilicity of the aromatic ring, while ortho- and para-substitution positions allow these groups to stabilize the Meisenheimer intermediate through resonance. In contrast, meta-substituents can withdraw electron density inductively but cannot provide resonance stabilisation, leading to markedly reduced exchange efficiency on the fluorine-19/fluorine-18 isotopic exchange. Overall, the apparent kinetic parameters indicated that strong electron-withdrawing substituents (NO₂, CN) promote more rapid isotopic exchange under the standardized reaction conditions employed, with a general trend of ortho ≥ para >meta in dimethyl sulfoxide.

Screening in dimethylformamide at 150 °C yielded generally lower conversions compared with dimethyl sulfoxide at 175 °C, and the positional trends observed in dimethyl sulfoxide were less pronounced. These results highlight the impact of solvent and temperature on exchange efficiency, suggesting that both stability and activation factors must be considered. As demonstrated by Blom et al. (Blom, Karimi and Långström 2009) and Shen et al. (2007), the choice of solvent can strongly influence the efficacy of isotopic exchange in aromatic substrates. Blom et al. reported that (pentafluorophenyl)(phenyl)methanone underwent more efficient fluorine-19/fluorine-18 exchange in dimethyl sulfoxide than in dimethylformamide, whereas Shen et al. found the opposite trend for benzaldehydes, with dimethylformamide providing higher incorporation.

Screening-scale experiments with 2-fluorobenzonitrile demonstrated that even at low substrate concentrations, the isotopic exchange reaction proceeded, theoretically allowing high molar activity. These screening reactions indicated the potential for clinically relevant molar activities. Scale-up experiments confirmed this feasibility: when 3.50 ± 0.25 GBq [^18^F]fluoride was used, the isolated product reached a molar activity of 121.4 ± 36.5 GBq/µmol.

### Limitations and outlook

The current work focused on simple aromatic systems, and kinetic fits for some substrates were poorly constrained. The high temperature and solvent employed may not be suitable for all substrates, as suggested by the instability observed for fluorobenzaldehydes. As all substrates were evaluated under standardized reaction conditions, the influence of reaction temperature on isotopic exchange efficiency was not investigated. The kinetic parameters reported here should therefore be regarded as apparent kinetic parameters that enable comparison between substrates under identical experimental conditions rather than intrinsic rate constants. The molar activity experiments were limited by the need to manually transfer the tetraethylammonium [^18^F]fluoride between vials, which restricted the maximum amount of radioactivity that could be used. By using a fully automated system, the reaction could be further scaled up by using larger amounts of starting radioactivity, which could afford higher molar activity. For the isolated 2-[^18^F]fluorobenzonitrile, the radiochemical yields and purities remained lower than desirable. Nonetheless, the results demonstrate that isotopic exchange can, under the appropriate conditions, deliver high molar activities without the need for separately functionalized precursors. By employing isotopic exchange, the development of new PET tracers with suitable electron-withdrawing substituents could be accelerated by bypassing the often lengthy process of precursor development, condition optimization, and purification method development.

## Conclusions

Together, these findings show that ortho and para electron-withdrawing substituents strongly promote isotopic exchange, while meta substituents hinder the reaction. Importantly, full-scale labeling of 2-fluorobenzonitrile yielded molar activities above 100 GBq/µmol, establishing isotopic exchange as a promising strategy for radiopharmaceutical labeling and motivating future work on more complex and clinically relevant scaffolds.

## Electronic supplementary material

Below is the link to the electronic supplementary material.


Supplementary Material 1


## Data Availability

Summary datasets are included in the published article, and raw data underlying graphical representations are provided in the supplementary information. Additional raw datasets are available from the corresponding author upon reasonable request.

## Abbreviations

PET: Positron emission tomography
HPLC: High-performance liquid chromatography
DMSO: Dimethyl sulfoxide
DMF: Dimethylformamide
