# Supplementary material for: The influence of functional groups and their relative position on isotopic exchange between 19F and 18F in aromatic rings
Source: EJNMMI Radiopharm Chem. 2026 Aug 31;11:63. doi: 10.1186/s41181-026-00494-4 (PMC13529628; doi:10.1186/s41181-026-00494-4)
Supplement: Supplementary file 1 [file 41181_2026_494_MOESM1_ESM.docx]

Supplementary Information for:

**The Influence of Functional Groups and Their Relative Position on Isotopic Exchange Between ¹⁹F and ¹⁸F in Aromatic Rings**

Antonia A. Högnäsbacka^1*^, Miguel A. Cortés Gonzalez^1^, Afrizki D. Wiranegara^1^, and Magnus Schou^1,2^

^1^Department of Clinical Neuroscience, Centre for Psychiatry Research, Karolinska Institutet and

Stockholm County Council, Stockholm, Sweden

^2^Radiology and Theranostics, Cancer Biomarker Development, Early Oncology R&D, AstraZeneca Stockholm, Sweden

**Table S1.** Chemicals and reagents used in this study.

| Compound | Supplier | Product code | Purity  [GC Area %] |
| --- | --- | --- | --- |
| 1,2-difluorobenzene | Sigma Aldrich | 126152 | 99.9% |
| 1,3-difluorobenzene | Sigma Aldrich | D102008 | 99.9% |
| 1,4-difluorobenzene | Sigma Aldrich | D102202 | 99.9% |
| 2-fluorobenzonitrile | Sigma Aldrich | 196649 | 99.8% |
| 3-fluorobenzonitrile | Sigma Aldrich | 235822 | 99.9% |
| 4-fluorobenzonitrile | Sigma Aldrich | 139416 | 99.4% |
| 1-fluoro-2-nitrobenzene | Sigma Aldrich | F10801 | 100.0% |
| 1-fluoro-3-nitrobenzene | Sigma Aldrich | 128392 | 99.9% |
| 1-fluoro-4-nitrobenzene | Sigma Aldrich | F11204 | > 99.9% |
| 2-fluorobenzaldehyde | Sigma Aldrich | F4807 | 99.9% |
| 3-fluorobenzaldehyde | Sigma Aldrich | F5005 | 99.8% |
| 4-fluorobenzaldehyde | Sigma Aldrich | 128376 | 98%* |
| fluorobenzene | Sigma Aldrich | F6001 | 100.0% |
| 1,2,3-trifluorobenzene | Sigma Aldrich | 324752 | 99.9% |
| 3-fluoroanisole | Thermo Scientific | A10931.09 | 99.6% |
| 4-fluoroanisole | Sigma Aldrich | F4602 | 99.4% |
| 2-fluorotoluene | Sigma Aldrich | F15323 | 99.7% |
| 3-fluorotoluene | Sigma Aldrich | 162329 | 99.9% |
| 4-fluorotoluene | Sigma Aldrich | F15331 | 99.9% |
| Sodium hydrogen carbonate | Sigma Aldrich | 1.06329 |  |
| Tetraethylammonium bicarbonate | Sigma Aldrich | 11268 |  |
| Acetonitrile (anhydrous) | Sigma Aldrich | 271004 |  |
| Dimethyl sulfoxide (anhydrous) | Sigma Aldrich | 5.89569 |  |
| *N,N*-Dimethyl formamide (anhydrous) | Sigma Aldrich | 227056 |  |

* Batch-specific CoA not available; purity according to supplier product specification.

**Figure S1.** 2-[^18^F]Fluorobenzonitrile analytical HPLC chromatogram showing the UV-trace and radiochemical purity of the isolated product. HPLC parameters: XBridge BEH C18 (150 x 4.6 mm, 5µm); 40% acetonitrile in 0.1M ammonium formate (aq.) at 2.0 mL/min and UV detection wavelength of 224 nm.

**Table S2.** Kinetic parameters for isotopic exchange of fluorobenzonitriles, nitrofluorobenzenes, and fluorobenzaldehydes in DMSO (175 °C). Apparent rate constants (k_obs_), half-lives (t_½_), and plateau conversions are reported with 95% confidence intervals.

| Compound | Position | K_obs_ (min^-1^) [95% CI] | t1/2 (min) | Plateau | R2 | n |
| --- | --- | --- | --- | --- | --- | --- |
| Fluorobenzonitrile | ortho | 1.14 [0.90-1.47] | 0.61 | 92 [89-96] | 0.97 | 3 |
|  | meta | 0.46 [0.20-1.19]^a^ | 1.52 | 68 [59-79] | 0.79 | 3 |
|  | para | 1.38 [0.83-2.55] | 0.50 | 84 [79-91] | 0.90 | 3 |
| Nitrofluorobenzene | ortho | 1.59 [1.45-1.74] | 0.44 | 94 [92-95] | 1.00 | 3 |
|  | meta | 0.69 [0.33-1.55]^a^ | 1.01 | 66 [59-75] | 0.82 | 3 |
|  | para | 1.41 [1.07-1.92] | 0.49 | 89 [85-93] | 0.96 | 3 |
| Fluorobenzaldehyde | ortho | 0.83 [0.39-1.90]^a^ | 0.83 | 71 [62-81] | 0.76 | 3 |
|  | meta | - | - | - | - | 3 |
|  | para | 0.97 [0.51-1.89] | 0.71 | 65 [59-71] | 0.86 | 3 |

ᵃ Wide CI; poorly constrained fit.

**Table S3**. Raw data from screening concentration-conversion dependency experiments with 2-fluorobenzonitrile. Starting activity, 2-fluorobenzonitrile concentration and radiochemical conversion are reported for each experiment.

| [^18^F]TEAF [MBq] | Concentration [µM] | Radiochemical conversion [%] |
| --- | --- | --- |
| 18.1 | 25000 | 89.97 |
| 18.2 | 25000 | 91.90 |
| 17.9 | 2500 | 77.13 |
| 18.1 | 2500 | 72.24 |
| 18.6 | 250 | 78.25 |
| 17.8 | 250 | 76.84 |
| 17.9 | 25 | 49.40 |
| 17.9 | 25 | 49.33 |
| 17.9 | 2.5 | 9.20 |
| 17.8 | 2.5 | 10.70 |

**Table S4.** Raw data from scale-up concentration-conversion dependency experiments with 2-fluorobenzonitrile. Starting activity, 2-fluorobenzonitrile concentration and radiochemical conversion are reported for each experiment.

| [^18^F]TEAF [MBq] | Concentration [µM] | Radiochemical conversion [%] |
| --- | --- | --- |
| 912 | 25000 | 69.54 |
| 629 | 250 | 34.43 |
| 965 | 25 | 11.47 |

**Table S5.** Raw data from molar activity experiments with 2-fluorobenzonitrile. Starting activity, isolated product activity, radiochemical purity, and product concentrations are reported for each experiment.

| [^18^F]TEAF [GBq] | Time (EOB) | Product [MBq] | Time (EOB) | Radiochemical purity [%] | Weight [g] | Concentration [nmol/mL] |
| --- | --- | --- | --- | --- | --- | --- |
| 3.3 | 0:38:25 | 127.3 | 01:13:36 | 87.09 | 0.78 | 1.31 |
| 3.787 | 0:39:32 | 256.9 | 01:14:12 | 92.97 | 0.76 | 1.56 |
| 3.421 | 0:41:36 | 115.8 | 01:17:27 | 85.59 | 0.79 | 0.83 |
